# Poly-Unsaturated Fatty Acids (PUFAs) from *Cunninghamella elegans* Grown on Glycerol Induce Cell Death and Increase Intracellular Reactive Oxygen Species

**DOI:** 10.3390/jof10020130

**Published:** 2024-02-04

**Authors:** Georgios Kalampounias, Chrysavgi Gardeli, Spyridon Alexis, Elena Anagnostopoulou, Theodosia Androutsopoulou, Panagiotis Dritsas, George Aggelis, Seraphim Papanikolaou, Panagiotis Katsoris

**Affiliations:** 1 Laboratory of Cell Biology, Division of Genetics, Cell and Developmental Biology, Department of Biology, School of Natural Sciences, University of Patras, 26504 Patras, Greece; tandroutsopoulou@ac.upatras.gr; 2 Laboratory of Food Chemistry and Analysis, Department of Food Science and Human Nutrition, Agricultural University of Athens, 75 Iera Odos, 11855 Athens, Greece; agardeli@aua.gr; 3 Hematology Division, Faculty of Medicine, School of Health Sciences, University of Patras, 26504 Patras, Greece; spirosal1@hotmail.com; 4 Laboratory of Food Microbiology and Biotechnology, Department of Food Science and Human Nutrition, Agricultural University of Athens, 75 Iera Odos, 11855 Athens, Greece; elena.anagn7@gmail.com (E.A.); spapanik@aua.gr (S.P.); 5 Unit of Microbiology, Division of Genetics, Cell and Developmental Biology, Department of Biology, School of Natural Sciences, University of Patras, 26504 Patras, Greece; dritsas.p@ac.upatras.gr (P.D.); george.aggelis@upatras.gr (G.A.)

**Keywords:** *Cunninghamella elegans*, cancer, cell lines, fatty acid lithium salts, gamma-linolenic acid, glycerol, polyunsaturated fatty acids, microbial lipids, oxidative stress, response surface methodology

## Abstract

*Cunninghamella elegans* NRRL-1393 is an oleaginous fungus able to synthesize and accumulate unsaturated fatty acids, amongst which the bioactive gamma-linolenic acid (GLA) has potential anti-cancer activities. *C. elegans* was cultured in shake-flask nitrogen-limited media with either glycerol or glucose (both at ≈60 g/L) employed as the sole substrate. The assimilation rate of both substrates was similar, as the total biomass production reached 13.0–13.5 g/L, c. 350 h after inoculation (for both instances, c. 27–29 g/L of substrate were consumed). Lipid production was slightly higher on glycerol-based media, compared to the growth on glucose (≈8.4 g/L vs. ≈7.0 g/L). Lipids from *C. elegans* grown on glycerol, containing c. 9.5% *w*/*w* of GLA, were transformed into fatty acid lithium salts (FALS), and their effects were assessed on both human normal and cancerous cell lines. The FALS exhibited cytotoxic effects within a 48 h interval with an IC50 of about 60 μg/mL. Additionally, a suppression of migration was shown, as a significant elevation of oxidative stress levels, and the induction of cell death. Elementary differences between normal and cancer cells were not shown, indicating a generic mode of action; however, oxidative stress level augmentation may increase susceptibility to anticancer drugs, improving chemotherapy effectiveness.

## 1. Introduction

Single-cell oils (SCOs) (*viz.* lipids produced by various types of microbial sources, i.e., yeasts, molds, algae, and bacteria) are hydrophobic compounds (mostly found in the form of triacylglycerols) that are accumulated to more than 20% *w*/*w* of total dry cell weight (TDCW) inside the cells or the mycelia of the lipid-producing microorganisms and are also called “oleaginous” [1,2,3,4]. SCOs present a great industrial and economic interest, since they can be employed as alternative lipid sources of expensive and rarely found into the Plant or Animal Kingdom lipids (i.e., SCOs can be employed as substitutes of fish oil, cocoa-butter, borage oil, evening primrose oil, etc.), as starting materials for the synthesis of the so-called “2nd generation” biodiesel, and as precursors in the (bio)-chemical synthesis of various types of oleochemical compounds [3,4,5]. Moreover, glycerol (glycerine), a renewable carbon source being generated in constantly increasing quantities as the main side-product of biodiesel producing facilities [3,6], has been used as substrate for SCO production by several oleaginous microorganisms [3,6,7].

Oleaginous Zygomycetes (fungi belonging to the species/genera including but not limited to *Mucor* sp., *Rhizomucor* sp., *Rhizopus* sp., *Mortierella isabellina*, *Cunninghamella echinulata*, *C. elegans*, *Thamnidium elegans*, *M. ramanniana*, etc.) have been employed as microbial cell factories amenable to producing mycelial mass containing, in varying concentrations, SCOs [2,3,4,7,8]. Lipids of these microorganisms, in most cases, contain unsaturated or poly-unsaturated fatty acids (PUFAs), whereas a PUFA that is found in different concentrations within the cellular lipids of these microorganisms is the fatty acid (FA) γ-linolenic (GLA—^Δ6,9,12^C18:3) [1,2,3]. It is noted that the γ- (and not the commonly found in several plant lipid commodities α-) isomer of this FA is a taxonomic characteristic for the Zygomycetes [1,9]. Lipids containing GLA are materials of very high added value for both the food and pharmaceutical industries, since they present anti-thrombotic, anti-irritant, and especially anti-tumor properties, and they are also effective in the treatment of various diseases such as inflammatory disorders, rheumatoid arthritis, and atopic eczema [1,9]. The (very few) vegetable oils that contain this FA are, in general, expensive, whereas, evidently, the potential of the design of a bioprocess through which significant quantities of fungal oils containing GLA can be obtained via fermentation/conversion of several low-cost substrates presents significant importance [2,4,7].

PUFAs are categorized into the ω-3 and the ω-6 families, with members from both families having been credited with potential cytotoxic activity against cancer cells. GLA is one of the strongest candidates of these FAs concerning the mentioned activity [10,11,12]. Proposed mechanisms of action vary; however, most findings suggest changes in the membranes’ ability to facilitate their transporting roles [13] and the elevation of oxidative stress levels that can stimulate the intrinsic apoptosis pathways and finally lead to cell death [14,15]. Additionally, interference with the regulation of the cell cycle has been reported, as has the activation of proapoptotic pathways [16,17,18]. Even though the mechanisms are not fully understood, another major problem restricting widespread research in the field is the administration routes. Dietary sources like seafood and plants have been the main mode of absorption; nevertheless, direct administration of GLA has shown increased cytotoxicity against various human cell lines and animal models, encouraging further research [19,20,21].

Because of the poor water solubility of FAs, their conversion into soluble forms is mandatory before their administration to cells or biological systems. A form of PUFAs that have not been intensively studied is fatty acid lithium salts (FALS), a water-soluble type of molecule that can be easily produced and remains stable at room temperature for extended periods. Administering FALS to patients dates back to 1998, when Kairemo et al. used lithium gamma-linolenate on patients with pancreatic cancer to investigate whether the permeability of cancer agents would increase after treatment, as well as the cytotoxic effects of GLA on the tumor [22]. FALS were also used by Ilc et al. in 1999, where purified GLA was utilized to examine the effects on glioblastoma cells [23]. However, due to the increased cost of lipid isolation and purification from their well-established sources, alternative ways to produce lipid preparations containing GLA are now available due to developments in microbial biotechnology, such as the previously mentioned potential utilization of Zygomycetes; for instance, Alakhras et al. in 2015 and Sayegh et al. in 2015 exploited Zygomycota strains to produce lipids rich in GLA and eicosapentaenoic acid (EPA), respectively, in the form of FALS and fatty acid potassium salts (FAPS) [24,25]. The lipid mixtures generated by the two groups had a high PUFA content, and when administered to cancerous cell lines (leukemia and breast cancer cells), cytotoxicity and DNA fragmentation were observed [24,25].

The purpose of this study was initially to study the dynamics of growth and lipid production by *C. elegans* NRRL-1393 during its growth on glycerol-based substrates in shake-flask nitrogen-limited experiments. Glucose was also employed, at the same initial concentration as glycerol, as a substrate. Previous preliminary trials of other Zygomycetes strains belonging our to collection (i.e., strains of the species *C. echinulata*, *C. elegans* and *Mucor circinelloides*) have demonstrated that the mentioned strain (NRRL-1393) showed the best results on glycerol employed as substrate, and for this reason it was chosen for further experiments. The transformation of lipids produced into FALS was studied and optimized, with process optimization being based on the response surface methodology (RSM) approach. Finally, the most important aim of the present study was to further expand the existing knowledge in the field by testing the effects of GLA-containing FALS on more cancerous cell lines. In conclusion, a non-extensively previously studied wild-type strain of *C. elegans* was grown on a cost-effective carbon source (glycerol), and the potential pharmaceutical effects of the derivatives of the produced SCOs were quantified, assessed, and studied in depth, in an approach of importance, for both its nutritional and pharmaceutical applications, with simultaneous benefits regarding sustainability and green economy.

## 2. Materials and Methods

### 2.1. Biological Material, Microbial Culture Media, and Coditions and Analyses Related to Microbial Growth

The microorganism used in the present study was the Zygomycete strain *C. elegans* NRRL-1393, kindly provided by the NRRL culture collection (Peoria, IL, USA). The strain was maintained on yeast peptone dextrose agar (YPDA), at a temperature of T = 4 °C and was subcultured every month to maintain its viability.

Experiments were performed in a semi-synthetic medium that had the following composition (g/L): KH_2_PO_4_, 7.0; Na_2_HPO_4_, 2.5; MgSO_4_·7H_2_O, 1.5; CaCl_2_, 0.15; FeCl_3_·6H_2_O, 0.15; ZnSO_4_·7H_2_O, 0.02; MnSO_4_·H_2_O, 0.06. The nitrogen sources used were (NH_4_)_2_SO_4_ and yeast extract, both at 0.5 g/L (besides its use as nitrogen source, yeast extract contains also valuable amino acids, peptides, vitamins, and micro-elements indispensable for microbial growth). Pure glycerol (purity ≈ 98% *w*/*w*) was used as a carbon source. Commercial glucose (purity ≈ 95% *w*/*w*) purchased from the Hellenic Sugar Industry (Thessaloniki, Greece) was employed as a “control” experiment. Both carbon sources were employed at an initial concentration of ≈60 g/L. Yeast extract (Honeywell Fluka) contained c. 8%, *w*/*w* nitrogen, and c. 12%, *w*/*w* carbon, while (NH_4_)_2_SO_4_ (Honeywell Fluka) contained 21% *w*/*w* nitrogen. By taking into consideration the carbon content of both glucose and glycerol (that is, ≈39–40% *w*/*w*), it may be assumed that the initial molar ratio C/N in the medium was ≈189–194 mol/mol. On the other hand, it is known that the level of de novo fatty acid synthesis is influenced by the nature and the initial quantity of the nitrogen sources [2,5], with the combination of ammonium sulfate and yeast extract resulted in the mentioned initial molar ratio C/N being quite common [1,5,7]. In contrast, although phosphate limitation (besides nitrogen) can also be used to trigger the process of lipid accumulation [1,2,5,7], preliminary experiments using reduced initial phosphate concentrations did not result in interesting mycelial biomass production by strain *C. elegans*, whereas equally the combination of initial KH_2_PO_4_, and Na_2_HPO_4_ concentrations of 7.0 and 2.5 resulted in satisfactory buffer capacity of the medium, with no significant pH fluctuations having been performed throughout the cultures realized. Therefore, trials in media using the previously mentioned salt composition of the medium were carried out under nitrogen-limited and carbon-excess conditions that have been formulated to favor the process of de novo lipid accumulation [2,4,7].

All the experiments were performed in 250 mL conical flasks, containing 50 ± 1 mL of growth medium, sterilized at T = 120 °C/20 min, and inoculated with 1 mL of spore suspension (around 1–3 × 10^5^ spores per flask). The pH value of the culture media after heat sterilization was 6.0 ± 0.1. All the cultures were incubated in an orbital shaker (New Brunswick Scientific, Edison, NJ, USA) at an agitation rate of 190 ± 5 rpm and an incubation temperature Τ = 28 ± 1 °C.

In the microbial fermentations, the analyses that were carried out dealt with the quantitative determination of fungal biomass, microbial lipids, and glycerol (glucose) consumed. Prior to any other analysis being performed, immediately after performing the selection of the flask that would constitute the experimental point, the dissolved oxygen (DO) concentration was quantitatively determined using a selective electrode (OXI 96, B-SET, Weilheim, Germany), according to a previously published procedure [26]. Oxygen saturation remained in values >30% (*v*/*v*) during all growth phases for both microbial substrates used (glucose and glycerol), indicating that the trials had been carried out under fully aerobic conditions [26,27], as it is already an established knowledge that an important prerequisite for sufficient de novo lipid production process is growth under sufficiently aerobic conditions into the growth medium [1,2,3,5]. Immediately after removing the flask from the incubator, the pH value of the culture medium was measured for all experimental points and all trials, by using a selective Jenway 3020 pH meter (Jenway, London, UK). The pH value of the medium did not significantly change regardless of the carbon source used, ranging in all fermentation steps from 5.1 to 5.7. Mycelia were collected through filtration under vacuum through Whatman No. 1 cellulose filter paper (diameter: 45 mm, thickness: 180 μm, fiber cut-off: 11 μm) and were extensively washed with distilled water. Total dry cell weight (TDCW) was determined through measurement of the dry matter of mycelia after placement of the wet biomass at T = 80 ± 2 °C until a constant weight was achieved (most usually this happened within 30 ± 4 h) [27]. Total cellular lipid (L, g/L) was extracted from the dried mycelia with a mixture of chloroform/methanol 2/1 (*v*/*v*) according to the procedure explicitly described by Sarantou et al. in 2021 [28], and was weighted after evaporation of the solvent. Lipids were converted to methyl-esters in a two-step reaction using consecutively methanolic sodium and hydrochloric methanol, and the produced fatty acid methyl esters were analyzed by the means of GLC according to Dritsas and Aggelis [27]. Finally, glycerol and glucose were quantitatively determined with the aid of High Performance Liquid Chromatography (HPLC) analysis, as indicated in detail in André et al. [29]. 

In all of the kinetic experiments performed, each experimental point presented in the figures and the tables is the mean value of two measurements deriving from independent experiments conducted at different times and with different inocula. The relative SE in most of the experimental points was ≤15%.

### 2.2. Preparation of C. elegans Fatty Acid Lithium Salts (FALS)

Fatty acid lithium salts (FALS) were prepared from the total of *C. elegans* lipids (192 h of fermentation) in a two-step procedure that involves the saponification step for fatty acid salts production, followed by the fatty acid’s reaction with LiOH, creating a fatty acid–lithium salt aqueous solution. The method is described in Alakhras et al. [24]. In brief, 1 g of microbial lipids was refluxed with 10 mL of a ΚOH 1M ethanol solution (95%). The aqueous layer was acidified with 10 mL of an HCl 4N solution and extracted with hexane (3 × 5 mL) in a separatory funnel. The emulsions formed in this step may be broken by adding an amount of salt (i.e., NaCl) to the separatory funnel. The free fatty acids (FFAs) were recovered after washing the organic phase repeatedly with water until neutrality, drying over anhydrous Na_2_SO_4_, and removing the hexane under vacuum. One gram of the received FFAs was diluted in 10 mL ethanol/diethyl ether (1:1, *v*/*v*) and LiOH 1N was added until the pH reached nine. After the removal of solvents under vacuum at 50 °C, H_3_PO_4_ 0.2N was gradually added to the soap solution under stirring until the pH reached seven. An approximately 10% *w*/*v C. elegans* FALS aqueous solution was prepared with the addition of distilled water to a final volume of 10 mL. 

### 2.3. Optimization of the Saponification Reaction of C. elegans Lipids

The RSM (a method of design and analysis of experiments) was employed to achieve the maximum possible fatty acid salts production (response) from microbial oil, minimizing prolonged reaction time, which favors the isomerization of lipids’ double bonds [30], reducing, if possible, the volume of hexane used, and standardizing the NaCl amount used for breaking down undesired emulsions. The design was constructed using three numeric variables, evaluating the optimum conditions for the saponification step. The variables tested were the reflux time (60–105 min), the volume of hexane used for the dissolution of the microbial oil (15–21 mL), and the amount of NaCl (1–3 g). A central composite design (CCD) was used, and a quadratic design model was performed. A total of 20 runs were determined by a selection criterion chosen during the experimental design. The Desing-Expert 11.0.5.0 (Stat-Ease, Inc., Minneapolis, MN, USA) was adopted for the RSM optimization study. The evaluation of the model’s fitness was confirmed using the *p*-values through an analysis of variance (ANOVA) and the determination coefficient (R^2^). The effectiveness of the saponification reaction in all runs was tested by analyzing the lipid extracts in a thin layer chromatography (TLC) plate to check the complete hydrolysis of acyl lipids. In brief, a spot of the poduced FFAs was applied on aluminum sheets coated in silica gel 60 (Merck, Darmstadt, Germany) (20 × 20 cm × 0.25 mm). The separation was carried out with n-hexane/diethyl ether/glacial acetic acid (70:30:1, *v*/*v*/*v*) and the visualization of lipid classes was accomplished in a tank of iodine vapor.

### 2.4. Determination of Fatty Acid Lithium Salts Content in Fatty Acids

For the determination (titration) of the free fatty acid (FFA) content of the produced FALS, 1 mL of the final product was acidified with 4 N HCl, followed by extraction in triplicate with hexane. Hexane was removed under vacuum at T = 50 °C and the residue was gravimetrically determined. The results were expressed as μg of FFAs per mL.

### 2.5. Cell Culture

The PC3 (ATCC) and DU-145 (ATCC) cell lines were used as human prostate carcinoma cell models. The cell lines TPC-1 (ATCC) and K1 (ATCC) were used as a papillary thyroid carcinoma cell model while the Nthy-Ori 3-1 cells (ATCC) were used to mimic normal thyrocytes. The cells were grown in RPMI 1640 medium supplemented with 10% fetal bovine serum (FBS), 100 units/mL penicillin, and 100 μg/mL streptomycin and were maintained at 5% CO_2_ and 100% humidity at 37 °C. Cell culture media (RPMI 1640 with stable glutamine, pyruvate, and NaHCO_3_) and cell culture-related reagents (fetal bovine serum, 0.25% trypsin solution in Phosphate Buffer Saline (PBS) and penicillin/streptomycin) were purchased from Biowest (Nuaillé, France) and Biosera (Nuaillé, France). Cell culture dishes, microplates, and transwell chambers were from Thermo Fisher Scientific (Waltham, MA, USA). Flow cytometry expendables and reagents were from BD Biosciences (Franklin Lakes, NJ, USA).

### 2.6. Proliferation Assays

Cells were seeded inside 48-well culture plates and cultured for 24 h. After this interval, the medium was aspirated, and fresh medium with increasing concentrations of FALS was added. A wide range of FALS concentrations were applied (9–135 μg/mL), and cells were incubated for 48 h inside the incubator. The media were aspirated, and the adherent cells (alive) were fixed with 4% formaldehyde in PBS for 15 min and then stained with 0.5% crystal violet in 25% methanol for 20 min [31]. Following gentle rinses with water, the microplates were left to air-dry, and the retained crystal violet was extracted using a 30% acetic acid aqueous solution. Afterward, the optical density at 595 nm was measured.

### 2.7. Migration Assays

The scratch test was used to assess the wound healing rate [32]. Cells were seeded inside 6-well microplates and left to grow until confluency. Using a sterilized P200 pipette tip (Greiner Bio-One, Kremsmünster, Austria), cruciform scratches were made, and the culture medium was aspirated. Fresh medium containing increasing concentrations of FALS was added, and photographs were taken using a microscope-mounted camera. The microplate was photographed at the key intervals of 0, 24, 48, and 72 h. The wound healing rate was calculated using the wound healing plugin for ImageJ v1.54h [33]. Migration, chemotaxis, and chemokinesis were assessed in Boyden chambers with 8 μm filters [34]. Serum-containing medium was added to the lower compartment (with or without FALS), and 2 × 10^4^ cells (suspended in plain RPMI 1640) were added into the insert. The cells were left to migrate for 24 h, and then the filters were fixed using a 4% *v*/*v* formaldehyde solution. Cells from the upper side of the filters were scraped, and the remaining (migrated) cells were stained using a 0.5% crystal violet solution. The filters were photographed under a microscope, and the percentage of migrated cells was calculated using the ImageJ software.

### 2.8. Fluorescence and Confocal Microscopy

Adherent cells were cultured on 16 mm glass coverslips inside 24-well microplates. The FALS-containing medium was added and left for 24 h. After this interval, the various staining procedures were performed. The coverslips were gently rinsed in a PBS solution and mounted on microscope slides using a Mowiol mounting medium. A Leica SP8 Confocal microscope and the LAS X software (version 3.0.16120.2) were used to view the samples, capture, and analyze all the photographs. Neutral lipid accumulation inside cells after incubation with FALS was visualized using the Nile Red (Sigma-Aldrich, Darmastadt, Germany) and BODIPY™ 505/515 (Sigma-Aldrich, Darmstadt, Germany) stains, by modifying protocols already used for microalgae [35] and lymphocytes [36]. The staining solution was added to the cells for 30 min. Nile red, bound to neutral lipids and sterols, is excited at 488 nm, and its maximum emission is at 565–585 nm [37,38]. Nile red bound to phospholipids is excited at 590 nm and emits light at 638 nm [36]. BODIPY™ binds to non-polar lipids and absorbs light at 493 nm, emitting it back at 503 nm. To visualize the cell membrane, the cells were stained with CellMask™ (Invitrogen™, Waltham, MA, USA) for 30 min, following the protocol provided by the manufacturer, and analyzed with a confocal microscope as previously described.

### 2.9. Flow Cytometry

The FACS Calibur (BD Biosciences, San Jose, CA, USA) was used to assess lipid accumulation, apoptosis, and intracellular reactive oxygen species levels. The adherent cells were incubated for specific time intervals in a FALS-containing medium, and after that, they were collected by trypsinization and centrifugation. The cell number was estimated using a Neubauer hemocytometer (Sigma-Aldrich, Darmstadt, Germany), and equal numbers of cells were used for the analyses. BODIPY™ and Nile red stains were utilized to quantify intracellular lipid content. Nile red was added (final concentration: 1 μg/mL) to the cells for 30 min at room temperature in the dark. Fluorescence channel 2 (yellow) was used to estimate neutral lipid and sterol content and fluorescence channel 4 (far red) was used for the estimation of phospholipids. A BODIPY™ staining solution was added to the cells with the same procedure as Nile red. Fluorescence channel 1 (green) was used to detect it. To assess apoptosis, cells were incubated in FALS-containing medium for 12, 24, and 48 h and then harvested as previously described. Afterwards, they were stained with propidium iodide and Annexin V-FITC for 15 min at room temperature in the dark [39]. To measure ROS, cells were stained with H_2_DCFDA (Sigma-Aldrich, Darmstadt, Germany) at 37 °C for 30 min following an already described procedure [40,41]. The subsequent analysis was performed with the FlowJo V10 software (BD Biosciences, San Jose, CA, USA).

## 3. Results

### 3.1. Growth of C. elegans on Glycerol—Comparisons with the Growth on Glucose

**Figure 1:**
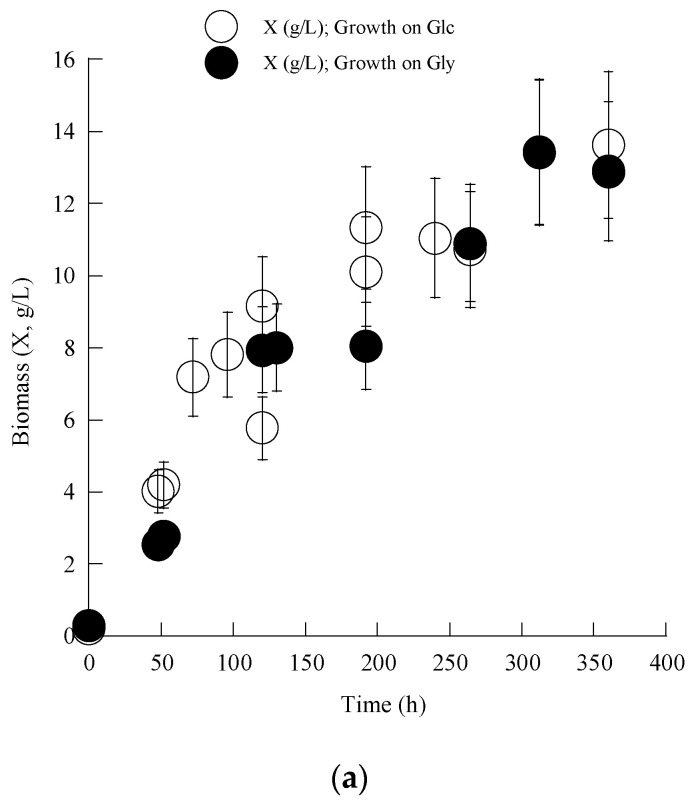

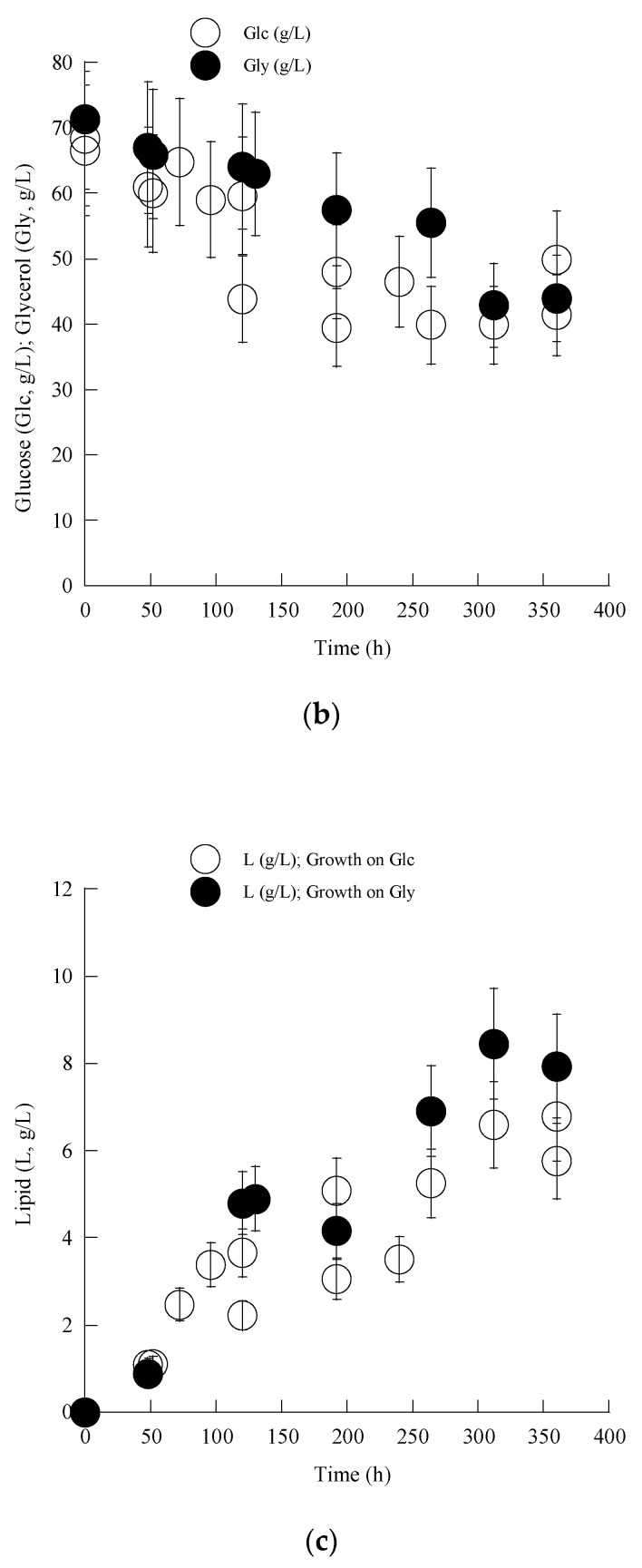
Growth of *Cunninghamella elegans* on glycerol/glucose. (**a**) Biomass (X, g/L) production; (**b**) substrate (Gly/Glc, g/L) assimilation and; (**c**) lipid (L, g/L) production during growth of *Cunninghamella elegans* NRRL-1393 on media composed of glucose or glycerol under nitrogen limitation in shake-flask experiments. Each experimental point is the mean value of two independent determinations (relative Standard Error (SE) for most experimental points ≤ 15%).

In the first part of this work, trials were performed on pure glycerol and commercial-type glucose used as carbon sources at an initial concentration (Gly_0_ or Glc_0_) adjusted to c. 60 g/L, while the simultaneous initial C/N molar ratio was c. 190 moles/moles. As indicated in the previous paragraphs, the fermentations were chosen to be performed under nitrogen-limited conditions, which are considered an important prerequisite to “boost” the cellular metabolism towards the de novo synthesis of microbial lipids [3,4]. The obtained results as regards the evolution of total dry cell weight (TDCW; X, g/L), substrate (glycerol; Gly and glucose; Glc, g/L), and lipid (L, g/L) are demonstrated in Figure 1a–c. 

From the obtained results, it can be seen that TDCW production was almost equivalent, irrespective of the trial perform on glucose or glycerol, since, in both instances, X_max_ concentration was achieved c. 330–360 h after inoculation, presenting similar values (≈13.5 g/L) for both trials (Figure 1a). Equally, the substrate assimilation rate was almost the same for the two substrates employed as carbon sources, in disagreement with most literature reports [3,29,42,43,44,45] that indicate that for most higher (e.g., Ascomycetes/Basidiomycetes) or lower (e.g., Zygomycetes) fungi, glucose is considered by far to be a more adequate carbon source compared to glycerol, due to the poor regulation of the enzymes implicated in the uptake of glycerol by these very microorganisms [29,43,44]. 

Moreover, it was with interest that, in the present investigation, significantly higher lipid production was observed to occur in the trial performed on glycerol (L_max_ = 8.4 g/L, simultaneous lipid in TDCW = 62.7% *w*/*w*), compared to the experiment performed on glucose (L_max_ = 7.0 g/L, simultaneous lipid in TDCW =51.4% *w*/*w*), demonstrating, once more, the suitability of glycerol as the implicated substrate in the conversions performed by *C. elegans*. 

### 3.2. Fatty Acid (FA) Composition of the Microbial Lipids Produced by C. elegans

The FA composition of the total cellular lipids (%, *w*/*w* of total lipids) of *C. elegans* produced during growth on glycerol and glucose for the beginning (t = 50 h), the middle (t = 192 h), and the end (t = 312 h) of the cultures is illustrated in Table 1. 

The obtained results demonstrate that the FA composition of the total cellular lipids (%, *w*/*w* of total lipids) of *C. elegans* does not present significant differences when growth is carried out on glycerol in comparison to the one performed on glucose. Lipids were mainly composed of oleic (^Δ9^C18:1; in concentrations ranging between 48% and 51% *w*/*w* of total cellular lipids), while these lipids were mostly unsaturated, and more unsaturated compared to the respective ones produced from oleaginous yeasts (see some state-of-the-art reviews, such as [1,2,3,4]). Other principal cellular fatty acids, found in lower concentrations in the lipids of *C. elegans* than the FA ^Δ9^C18:1, were the FAs linoleic (^Δ9,12^C18:2), palmitic (C16:0) and the important as regards its pharmaceutical actions GLA (^Δ6,9,12^C18:3). The FA composition of the *C. elegans* lipids presented small differentiations as a function of the fermentation time for both the experiments performed on glucose or glycerol, with the FA ^Δ9^C18:1 presenting a small increase and the FA ^Δ6,9,12^C18:3 a slight decrease with the time, suggesting that the rate of dehydrogenation of the FA ^Δ9,12^C18:2 into ^Δ6,9,12^C18:3 is slightly lower than the rate of lipid accumulation in this microorganism. A potential increase in the agitation rate of the culture could even more somehow increase the unsaturated FA content of cellular lipids, since it is known that this unsaturation process is related with the higher oxygen concentration found into the medium [1,2,3,5].

As mentioned in the previous paragraphs, Zygomycetes in general produce, inside their cellular lipids, mostly the γ- isomer of the FA linolenic (^Δ6,9,12^C18:3). This FA presents interest, due to its nutritional and pharmaceutical significance [1,3]. The lipids of some plants that contain γ-linolenic acid are currently commercialized (this is the case of the oil deriving from the plant *Oenothera biennis* that contains this FA in concentrations of 8–10% *w*/*w*) [1,2]. In the current investigation, the quantities of γ-linolenic acid produced were similar to the ones of the lipids of *O. biennis* (Table 1). Moreover, the maximum quantity of GLA produced by *C. elegans* in the present study was ≈525 mg per L of medium for the experiment in which the fungus was grown on glucose. On glycerol, the respective value was c. 490 mg per L of medium. The highest quantities of γ-linolenic acid that have been produced by wild-type Zygomycetes are recorded by *C. echinulata* ATHUM 4411, *C. echinulata* CCRC 31840, and *Thamnidium elegans* CCF-1465 (quantities ranging between 900 and 1200 mg/L of medium) [46,47,48]. 

### 3.3. Optimization of the Saponification Reaction of C. elegans Lipids

**Figure 2:**
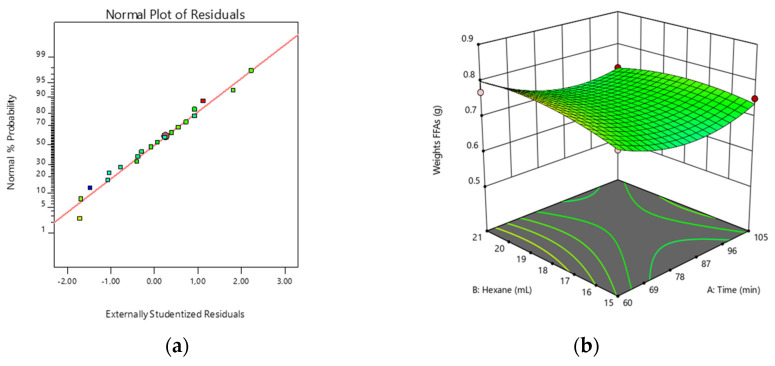
Optimization of the saponification reaction. (**a**) Normal plot of residuals and (**b**) response surface and contour plot indicating the effect of reflux time (min) and volume of hexane (mL) on FFA production (g).

Our experimental data of FFAs production (response factor) using three variables (the reflux time, the volume of hexane, and the amount of NaCl) ranges between 0.6722 g and 0.8732 g (Table A1). The results of the ANOVA demonstrate a non-significant lack of fit (R^2^ of 0.82) which describes the fitness of the data predicted by the model. The F-value (4.96) of the model implies that the model is significant; there is only a 0.99% chance that an F-value this large occurs due to noise. *p* values of less than 0.05 indicate that the model terms are significant. In this case, A^2^ and B^2^ are significant model terms that affect FFA production. Figure 2a demonstrates the probability plot between the studentized residual and percent probability of response that confirms data homogeneity. Data points should be approximately linear. A non-linear pattern (such as an S-shaped curve) indicates non-normality in the error term, which may be corrected by a transformation. Figure 2b demonstrates a 3D surface plot with its contour lines, which indicates that the least reflux time of 60 min results in the highest FFA production.

To construct desirability indices, the goal was to minimize the reflux time. Desirabilities range from zero to one for any given response. A value of one represents the ideal case. The optimum conditions and desirability of the response are presented in Table 2.

The optimum conditions proposed were less time-consuming than those described in Alakhras et al. [24] (60 min of reflux time), whereas the use of 3 × 5 mL of hexane (B) and 1 g of NaCl (C) was confirmed, maximizing the desirability index to 0.849.

### 3.4. C. elegans Fatty Acid Lithium Salts Inhibit the Growth of Normal and Cancerous Cell Lines

**Figure 3:**
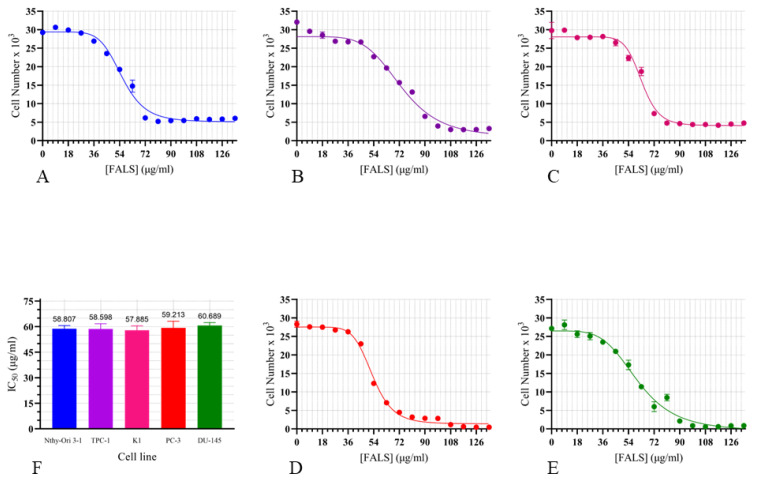
Proliferation assays. Following 48 h of incubation with increasing concentrations of *Cunninghamella elegans* FALS, the number of remaining alive cells was determined using the crystal violet assay. The data were analyzed using GraphPad Prism 8. Proliferation curve of: (**A**) Nthy-Ori 3-1, cells; (**B**) TPC-1 cells; (**C**) K1 cells; (**D**) PC-3 cells; and (**E**) DU-145 cells. All experiments were conducted in triplicate, and every point represents the average cell number ± standard error of the mean (SEM) values. (**F**) Using the built-in equations of Prism 8, the IC_50_ was calculated. Statistical analysis with a one-way ANOVA test did not reveal any significant differences between the cell lines that were monitored. The average IC_50_ was determined to be 58 μg/mL.

The incubation with fatty acid lithium salts from *C. elegans* resulted in cytotoxic effects following a dose–response model in every cell line used. The Nthy-Ori 3-1 cell line was used as a normal thyrocyte model, while the cell lines TPC-1 and K1 were used as models of thyroid carcinoma. Additionally, the well-established prostate cancer cell lines PC-3 and DU-145 were used as models of cancerous epithelial cells from a different origin. The cells were incubated for 48 h with a wide range of FALS concentrations to study the susceptibility of each cell type and determine the half-maximal inhibitory concentration (IC_50_) (Figure 3). The effects of lithium (in the form of LiOH diluted in PBS) in the same range of concentrations were also assessed, to clarify whether the cytotoxicity observed was an effect of the fatty acids or the lithium cations that have known antitumor properties. These experiments indicated that lithium did not affect cell viability or the proliferation rate when used at concentrations similar to those of FALS experiments (i.e., 0.05–0.5 mΜ) (Appendix B).

The IC_50_ of each cell line was calculated using the built-in tools of the Prism 8 software (GraphPad, San Diego, CA, USA). Each experiment was repeated at least three times, and the mean IC_50_ value alongside the SEM appears in Table 3.

Following analysis with a one-way ANOVA test, no significant differences were observed among the cell lines that were examined (*p* = 0.8787). Therefore, we selected an approximate concentration at which every cell line exhibited cytotoxicity to investigate further effects on other cell functions. The selected FALS concentration was 54 μg/mL, which was tolerated by all cell lines and allowed us to treat the cells with FALS for an extended period of time. We managed to maintain a clone of each cell line in the constant presence of 54 μg/mL FALS, and this clone was designated as fattened. Subsequently, we focused on the cancerous cell lines PC-3 and DU-145 to study more aspects of the FALS anticancer potential. These specific cell lines are derived from metastatic cells of prostatic origin, and they are commonly used as cancer models for drug development because they maintain a high proliferation rate and metastatic potential.

### 3.5. C. elegans Fatty Acid Lithium Salts Decrease the Migration and Wound Healing Ability of Cancer Cells

**Figure 4:**
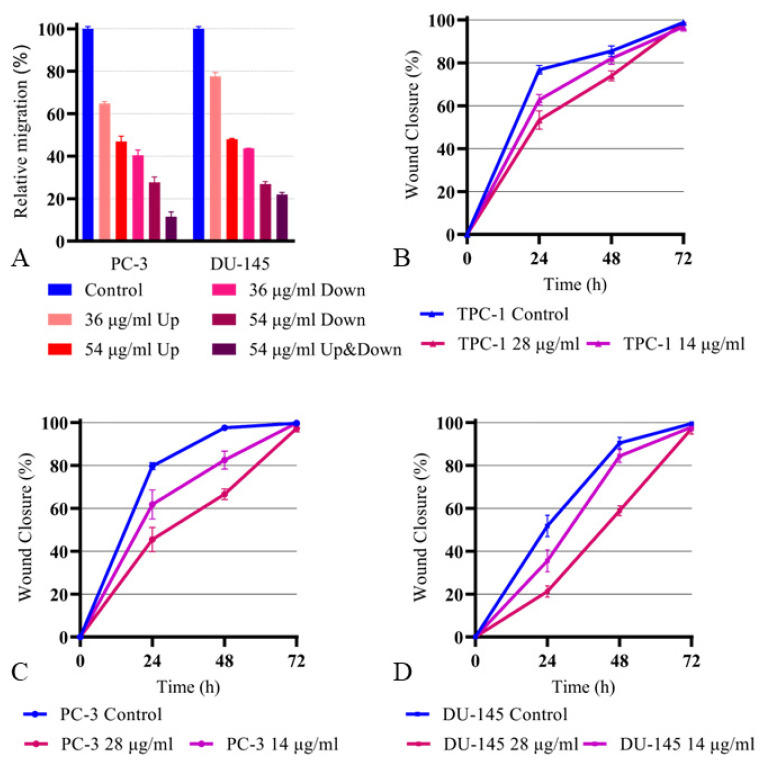
Migration assays. (**A**) Using Boyden chambers, we assessed the effects of *Cunninghamella elegans* FALS on PC-3 and DU-145 cells after incubation for 24 h. A culture medium with 20% FBS was used as a control sample. To assess chemotaxis, FALS were added to the chemoattractant medium (lower compartment), while, to assess migration, FALS were present in the cell medium (upper compartment). Chemokinesis was assessed by supplementing both compartments with FALS. After the designated time interval, the cells were fixed, stained with crystal violet, and photographs were taken using a photonic microscope. The images were analyzed using the built-in tool cell counter by ImageJ. The data analysis was performed on Prism 8. (**B**) Using the scratch test, we assessed the effects of FALS on the wound healing ability of TPC-1 cells. Low doses (14 μg/mL and 28 μg/mL) of FALS were added after the wound formation, and photographs were taken at the key time points of 0, 24, 48, and 72 h. Cells supplemented only with 10% FBS were used as control samples. The data analysis was performed using the wound healing macro tool in the ImageJ software, and all the graphs were designed using Prism 8. The same procedures were applied to (**C**) PC-3 cells and (**D**) DU-145 cells.

The effects of FALS on the migration, chemotactic motility, and wound healing rate of the cancerous cell lines PC-3 and DU-145 were assessed. Following incubation with high (54 μg/mL) and moderate (36 μg/mL) FALS concentrations, the cell migration rate seems to be decreased, as was demonstrated using the transwell/Boyden chambers assay (Figure 4A). 

The chamber configuration was used for 24 h, and as a chemoattractant, a medium containing 20% FBS was added in the lower compartment. Three configurations were tested to assess: (a) the effect of FALS on the cell migratory rate (FALS were added in the upper compartment); (b) the possible chemoattractant role of FALS (they were added in the lower compartment); and (c) the effects of FALS on the chemokinesis (equal FALS concentration was present in both compartments). A moderate concentration of FALS (36 μg/mL) inhibited cell migration of both cell lines (~20% in the DU-145) while the higher dose of 54 μg/mL reduced their migratory potential by 50%. The addition of FALS inside the chemoattractant medium reduced chemotactic movement by 60% (36 μg/mL FALS) and by 75% once the FALS dose was elevated (54 μg/mL). Regarding chemokinesis, both cell lines exhibited a dramatic drop in motility, which was about 90% for the PC-3 cell line and 80% for the DU-145 cell line (cultured in a 54 μg/mL FALS concentration). 

The FALS were also tested on the wound healing abilities of TPC-1, PC-3, and DU-145 cells and exhibited dose-dependent inhibitory effects. Lower doses of FALS were used (14 μg/mL and 28 μg/mL) because of the extended duration of the assay (72 h). The wound healing ability of all three cell lines was impaired, as the wound closure in FALS-treated samples was observed to close slower than the respective control samples containing medium with 10% FBS. The selected concentrations allowed full healing of the scratches within 72 h (Figure 4B–D); however, higher concentrations completely inhibited cell motility and even reduced the final live cell number due to cytotoxic effects. A more detailed explanation of the migration experiments, as well as the initial photographs, are presented in Appendix B. 

### 3.6. Fatty Acids from C. elegans Fatty Acid Lithium Salts Are Stored Inside the Endoplasmic Reticulum

**Figure 5:**
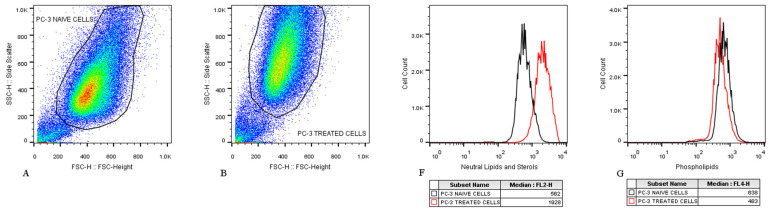

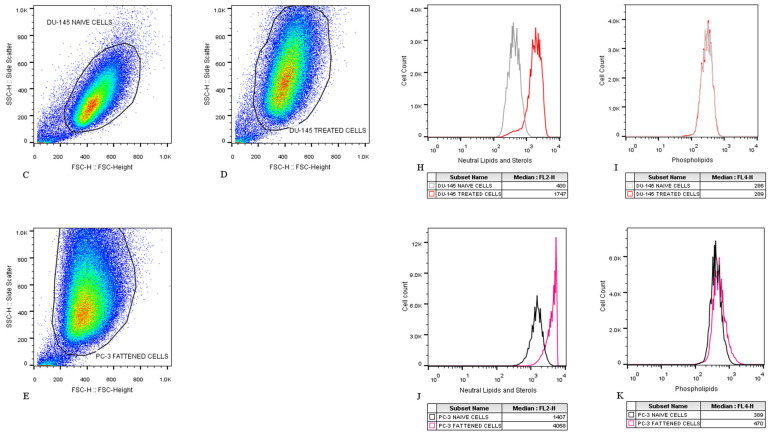
Lipid accumulation examination using flow cytometry. Following incubation with 54 μg/mL FALS for 24 h, cells were trypsinized and analyzed with flow cytometry to observe changes regarding shape and size. (**A**) Untreated PC-3 cells and (**B**) treated PC-3 cells exhibited differences mainly regarding complexity, as shown by SSC data. (**C**) The untreated DU-145 cell population was more concentrated than the (**D**) treated DU-145 cells. Treated cells were bigger, more diverse, and generally more complex. (**E**) The fattened PC-3 cells had also increased complexity and heterogeneity. (**F**) By staining the cells with Nile red, the lipid accumulation was verified. Using the yellow fluorescence channel (FL:2), an accumulation of neutral lipids was detected in the PC-3 cell line (red line), a result also evident in the DU-145 cells (red line) (**H**). (**G**,**I**) Using the far-red fluorescence channel (FL:4), the phospholipids were assessed and did not indicate any changes during the initial 24 h following FALS administration. (**J**) The fattened cells (magenta line) exhibited maximum lipid accumulation following several weeks of constant FALS presence, while (**K**) their phospholipid content was almost unchanged.

The administration of FALS was documented to change the cell morphology after microscopic observation (Appendix C). The cells were found to have atypical shapes, and they appeared darker under the microscope. The majority were slightly enlarged, and granules were seen inside their cytoplasm. This morphology was also verified by flow cytometry; all treated cells had increased size (compared to the untreated clones) as indicated by forward scatter data, as well as increased complexity as defined by side scattering (Figure 5A–E).

To examine whether the altered morphology was a result of induced cell death, membrane deformities due to the surfactant FALS nature, or the accumulation of fatty acids inside the cells, we stained them with the fluorescent dyes CellMask™, Nile red, and BODIPY™ 505/515. CellMask™ staining does not penetrate the cell membrane of alive cells, and it is ideal for imaging the cell surface. Our experiments did not reveal visible membrane alterations or deformities, aside from the enlarged shape, which was already visible by ordinary microscopes (Appendix C). 

**Figure 6:**
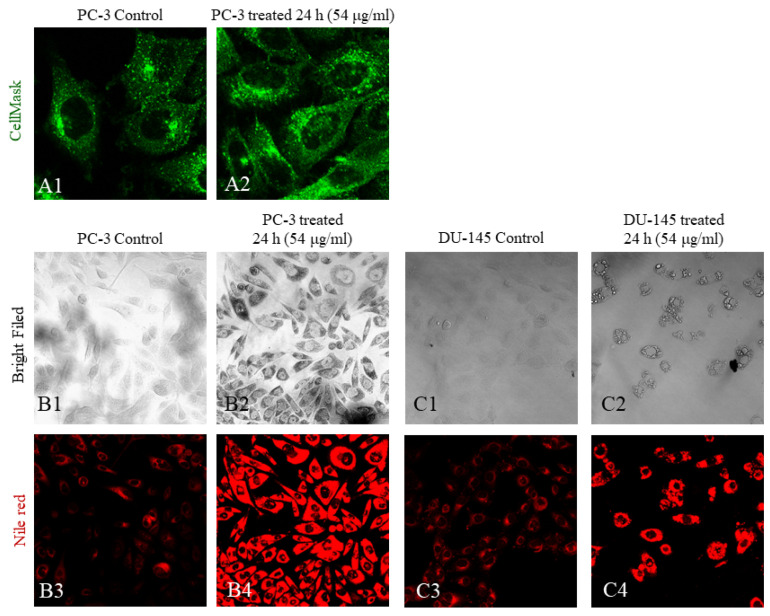
Cell morphology imaging using CellMask™ and Nile red. Following incubation with 54 μg/mL FALS for 24 h, cells grown on glass coverslips were stained and visualized under a confocal microscope. (**A1**) Untreated PC-3 cells (magnification ×630) and (**A2**) treated PC-3 (magnification ×630) did not exhibit any visible membrane deformities following treatment with FALS; (**B1**) bright-field image of untreated PC-3 cells (magnification ×400) and (**B2**) image after incubation with FALS (magnification ×400); (**B3**) staining of untreated PC-3 cells with Nile red. The excitation was performed at 488 nm, and the emission at 565 nm was captured. The dim fluorescent intensity is due to the staining of intracellular lipids in the endoplasmic reticulum (ER); (**B4**) treated PC-3 cells exhibit bright fluorescence because of intracellular lipid accumulation in the ER (magnification ×400). (**C1**) Regarding cell morphology, analogous observations were made in the DU-145 cell line (magnification ×400), where granulation was amplified in the treated cells (magnification ×400) (**C2**); (**C3**) the untreated DU-145 cells exhibited dim fluorescence in basal conditions (magnification ×400), while; (**C4**) the treated cells had granulous stained areas, which are thought to be lipid droplets inside the ER (magnification ×400).

Examination with a fluorescent microscope after staining with BODIPY™ revealed that the treated cells had increased fluorescence intensity because of lipid accumulation (Appendix D). Nile red staining offered better distinguishment among the various cell structures, and it was also used to visualize intracellular structures by confocal microscopy. Nile red can emit fluorescence at a wide range of wavelengths, depending on the hydrophobic molecule upon which it is bound. Phospholipids and neutral lipids can be distinguished, and this property allowed us to quantify the different lipids. Confocal microscopy revealed great differences between untreated and treated cells, even after only 24 h of incubation. Structures known as lipid droplets were observed, verifying the active accumulation of the fats during that interval (Figure 6(B2,B4,C2,C4)). Furthermore, the fattened cells were observed to completely regain their typical morphology 36–48 h after FALS withdrawal. Flow cytometry revealed that the accumulated fats inside the cells were neutral lipids rather than phospholipids. Additionally, it was demonstrated that the phospholipid content of untreated and treated cells was almost identical in most cases, while for fattened cells, it was slightly elevated compared to both clones (Figure 5F–K). 

### 3.7. C. elegans Fatty Acid Lithium Salts Can Induce Cell Death

**Figure 7:**
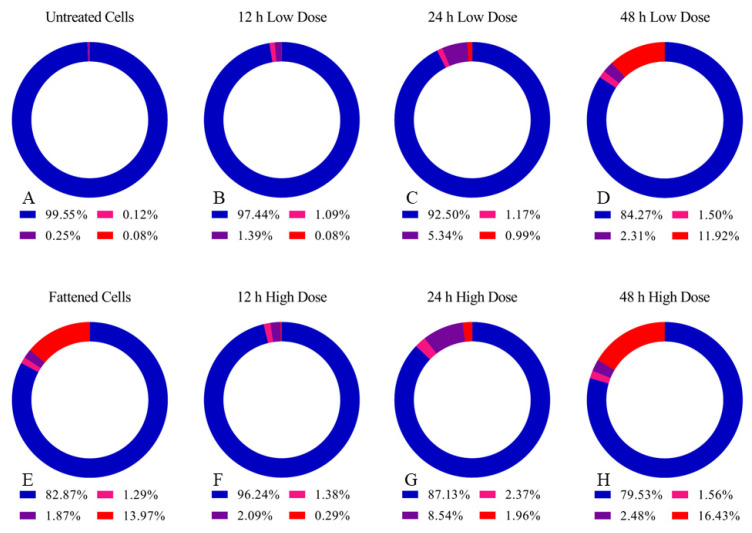
Apoptosis assays using Annexin V and propidium iodide (PI). Cells were incubated with moderate/low (36 μg/mL) and high (54 μg/mL) doses of *Cunninghamella elegans* FALS and then trypsinized. The apoptotic/necrotic and ferroptotic portions (of detached cells) were analyzed with flow cytometry. The blue ribbon corresponds to viable cells (Annexin V-/PI-), the purple ribbon to early apoptotic cells (Annexin V+/PI-), the magenta ribbon to late apoptosis (Annexin V+/PI+), and the red ribbon to necrotic/ferroptotic cells (Annexin V-/PI+). (**A**) As a control sample, we used alive untreated PC-3 cells with a stable rate of apoptosis of about 0.5%. (**B**–**D**) The low FALS dose effects were assessed at 12, 24, and 48 h, and loss of cell viability down to 84% was documented; (**F**–**H**) the high FALS dose effects were also assessed at 12, 24, and 48 h and were found to decrease cell viability by 21%. (**E**) The fattened cells were found to have a great portion of non-viable cells, despite the constant presence of FALS.

To study whether the cell death caused after incubation with high FALS doses was apoptosis, we used flow cytometry and the Annexin V-FITC/propidium iodide assay. Annexin V-positive cells are apoptotic. Annexin V-negative cells are either necrotic or ferroptotic (once they are propidium iodide (PI)-positive), and double-negative cells are live cells. Double-positive cell populations are viewed as cells in late apoptosis, while Annexin V single-positive cells are in early apoptosis. Herein, we compare the percentage of cells currently undergoing cell death, although the numbers concerning cell viability in reality are even lower since only the attached cells are measured. Following treatment with FALS for 12–48 h, a significant portion of the cells underwent some kind of cell death of apoptotic or non-apoptotic nature (Figure 7).

Regarding the moderate FALS dose of 36 μg/mL, the percentage of alive cells dropped from 99.5% to about 84% after 48 h of FALS presence (Figure 7A–D). Cells treated with a higher FALS dose (i.e., 54 μg/mL), close to the 48 h IC_50_, exhibited decreased viability (~85% after 24 h), with the majority of non-viable cells being negative for Annexin V and positive for PI (Figure 7F–H). Cells negative for Annexin and positive for PI are believed to undergo necrotic or ferroptotic death, which is also consistent with the PUFAs effects. This was evident in all of our experiments and exhibited a dose-dependent pattern considering the FALS dose. The fattened clone was constantly treated with FALS, and the cell medium was replaced every 72 h. During the time of the measurement, the double negative population accounted for ~80% of the total cells, a great portion was necrotic, and small percentages (1–2%) were found to be currently undergoing apoptosis (Figure 7E). The fattened cells had lower Annexin V-/PI+ percentages, possibly indicating some kind of adaptation to the toxic conditions imposed by the elevated FALS presence. The flow cytometry data are presented in Appendix E.

### 3.8. Administration of C. elegans Fatty Acid Lithium Salts Increases the Levels of the Intracellular Reactive Oxygen Species (ROS)

**Figure 8:**
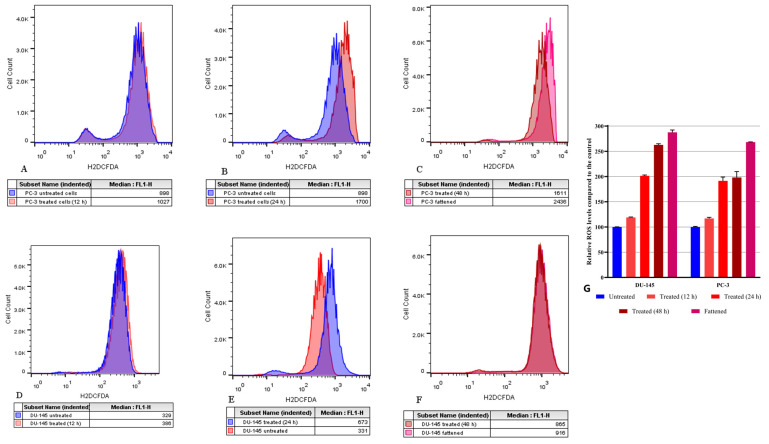
Intracellular reactive oxygen species (ROS) determination with flow cytometry using the H_2_DCFDA assay. Cells were incubated with high doses of *Cunninghamella elegans* FALS (54 μg/mL) and then trypsinized. The histograms represent different time points where ROS were measured in both cell lines. (**A**) Untreated PC-3 cells and cells treated for 12 h; (**B**) PC-3 cells treated for 24 h; and (**C**) PC-3 cells treated for 48 h compared to fattened PC-3 cells. (**D**) Untreated DU-145 cells and cells treated for 12 h; (**E**) DU-145 cells treated for 24 h; and (**F**) DU-145 cells treated for 48 h compared to fattened DU-145 cells. (**G**) Following analysis with Prism 8, the results are presented as relative ROS accumulation compared to the control group. All experiments were performed in triplicate, and the results were normalized using the untreated population of each cell line. The values represent averages ± SEM. Statistical analysis with a one-way ANOVA test revealed significant changes among all groups (*p* < 0.0001).

Finally, we assessed the effects of FALS on cellular oxidative stress levels. Unsaturated fatty acid metabolism is a major source of free radicals, mainly in the form of lipid peroxides. Incubation with high FALS doses (54 μg/mL) rapidly elevates ROS levels. Following 12 h of FALS administration, the generated ROS levels increased by ~20% in both cell lines, while, after 24 h, a 90% increase was documented in the PC-3 cell line and a 100% increase in the DU-145 cell line, respectively (Figure 8A,D).

After 48 h of continuous FALS presence, the PC-3 cell line had almost identical ROS levels with the 24 h time point, indicating a plateau, while the DU-145 had increased intracellular ROS levels by 160% compared to the control (Figure 8B,C,E,F). We also assessed the fattened clones for ROS levels, and we measured an increase of over 170% in both cell lines, confirming the amplified ROS generation resulting from a PUFA-rich medium (Figure 8C,F). The percentages are summarized in Figure 8G, where the relative ROS accumulation is presented compared to the untreated cell clone of each cell line.

## 4. Discussion

*C. elegans* NRRL-1393 produced significant TDCW quantities during the growth of glycerol or glucose under nitrogen-limited conditions, in fermentations performed in shake-flask trials. When growth was performed on glycerol, the production of biomass was similar to that observed during growth on glucose, while the assimilation rate of these compounds was also similar. Moreover, slightly higher lipid production occurred in the glycerol-based media compared to the trials performed on glucose. This is, as mentioned, quite surprising as a result. According to the contemporary literature, glycerol in general is not a very appropriate substate amenable to be used for most fungi (higher or lower) due to its poor uptake regulation. For most higher or lower fungi, growth and lipid production on glucose is significantly better than that on glycerol; for instance, the growth of fungi of the genera *Aspergillus*, *Lentinula*, *Mucor*, *Morchella*, etc., on glycerol was accompanied by lower dry biomass production compared to growth on glucose [27,29,42,43,49]. This is not the case with the growth of oleaginous yeasts (i.e., *Rhodosporidium toruloides*, *Yarrowia lipolytica*, *Debaryomyces prosopidis*, etc.) that in general present excellent growth on glycerol [26,28,50]. A noticeable exception to this rule is the Zygomycete *Thamnidium elegans* as well as some strains of macrofungi (i.e., *Ganoderma lingzhi, Grifola frondosa*) that presented, indeed, very interesting microbial growth on media composed of glycerol, almost similar to the one obtained on glucose [49,51]. 

Cellular lipids of *C. elegans* presented similar total FA composition during the growth of the fungus on glycerol- or glucose-based media. This agrees with the results reported for several oleaginous Zygomycetes (e.g., *Mucor* sp., *Rhizomucor pusillus*, *Umbelopsis isabellina*, *C. echinulata*, etc.) cultivated on renewable sugar- or glycerol-based substrates under nitrogen-limited conditions [8,27,42,45,52]. Moreover, as mentioned in the previous paragraphs, Zygomycetes in general produce inside their cellular lipids mostly the γ- isomer of the FA linolenic (the GLA; ^Δ6,9,12^C18:3, belonging to the ω-6 series) instead of the α- one (*viz.* the alpha-linolenic acid—ALA; ^Δ9,12,15^C18:3, belonging to the ω-3 series), the latter being mostly produced in yeasts and higher fungi [1,2,7]. As also was previously indicated, the lipids of some plants that contain γ-linolenic acid (this is a rare situation, since most candidates of the plant kingdom produce lipids that contain the ALA isomer; see: [1,7]) are currently commercialized (this is the case for the oil derived from the plant *Oenothera biennis*, also called evening primrose oil, that contains this FA in concentrations of 8–10% *w*/*w*) and their cost is estimated to be USD45–50 per kg [1,2]. Moreover, the maximum quantity of GLA produced by *C. elegans* in the present study was ≈525 mg/L during growth on glucose, and *c*. 490 mg/L during growth on glycerol. These results, although quite interesting, compare somehow unfavorably with the highest quantities of γ-linolenic acid that have been reported by wild-type Zygomycetes, which are found within the magnitude of 900–1400 mg/L. For instance, maximum GLA concentrations that have been recorded by *C. echinulata* ATHUM 4411 were =1018 mg/L [47], by *C. echinulata* CCRC 31840 = 964 mg/L [53] and =1349 mg/L [46], while by *Thamnidium elegans* CCF-1465 were =1014 mg/L [48]. Therefore, further experiments related to the optimization of GLA quantity by *C. elegans* would potentially be needed in the future. 

In this study, lipids from *C. elegans* were administered for the first time to human cancer models, and their anti-proliferative, anti-migratory, and stress- and cell death-inducing effects were observed. *C. elegans* was grown on industrial-grade glycerol, a byproduct of the food and soap industries, and in nitrogen-deprivation conditions, it accumulated polyunsaturated lipids rich in GLA, a molecule with well-established anti-cancer activity [11,54]. Previous publications have reported GLA’s capability to suppress cell proliferation and induce apoptosis [55]; however, only one study has used microbial lipids (ML)s containing it, and observations have only been made on the leukemia cell line HL-60 [24]. For the first time, we used solid tumor models of thyroid and prostate cancer to study the same phenomenon, and confirmatory results arose. 

The models of our study exhibited similar IC_50_ values (~56 μg/mL) following 48 h of incubation with FALS. The normal thyrocyte model Nthy-Ori 3-1 did not exhibit significant differences compared to its cancerous counterparts K1 and TPC-1 concerning IC_50_; nevertheless, more research is needed on the topic since the effects could have a long-term signature. Additionally, normal thyrocytes are physiologically under the control of TSH, which regulates their mechanism, a function that was not emulated in our study. The prostate cancer cell lines PC-3 and DU-145 were also susceptible to FALS-caused cytotoxicity following a dose-dependent decrease in cell viability following incubation with the salts. Additionally, in this study, we examined for the first time the effects of GLA-rich MLs (in the form of FALS) on cancer cell migration. By introducing moderate and low doses of *C. elegans* FALS, a decrease in cell motility was observed, as well as a decrease in chemotactic movement and wound healing ability, observations that have also been made in cancer cells after treatment with pure GLA [56]. The same phenomenon was evident in both prostate and thyroid cancer cells, with both of them having high metastatic potential under basal conditions.

Alakhras et al. (2016) observed synergistic actions between H_2_O_2_, which is a ROS source, and *C. echinulata* FALS [24]. *C. echinulata* and *C. elegans* both produce PUFAs high in GLA, and the latter has already been correlated to the accumulation of ROS and lipid peroxidation [20,57,58]. Our study measured the direct elevation of intracellular ROS levels following treatment with FALS and found them to be significantly elevated. This explains the observed synergistic effects by previous groups and also verifies the subsequent cell death, which is a result of intrinsic apoptosis pathway activation [59]. 

Our assays demonstrated an induction of both apoptotic and non-apoptotic death in treated cells in the form of an increase in both Annexin V-positive and propidium iodide-positive populations. The rapid elevation of intracellular oxidative stress levels is believed to be the trigger mechanism not only of apoptosis but also of the iron-dependent death pathway of ferroptosis [60]. Ferroptosis is thought to be affected by PUFA metabolism, and a key point of its regulation is the peroxidation of the cell membranes [61,62,63]. Accumulation of PUFAs inside the cells has been considered to increase cell susceptibility to lipid peroxidation, especially when antioxidant agents such as tocopherols and carotenes are absent, like in our culture conditions [64,65]. Sensitization to ferroptosis induction could be an effective way to overcome drug resistance, given this emerging problem from the use of chemotherapies [66]. In our study, we maintained cell clones for extended periods in FALS-containing medium, and we were able to show the stable accumulation of neutral lipids on the inside of the cells as lipid droplets. Excessive amounts of intracellular lipids were verified to be stored using Nile red and confocal microscopy, and this accumulation is believed to have caused significant changes in the cell metabolism, causing an amplification of beta-oxidation, the main route of lipid degradation and a major source of free radicals [67]. In vivo studies on rats have shown that long-term consumption of ω-6 PUFAs causes metabolic alterations that elevate ROS and apoptosis levels, an observation also made by us on the fattened clone of DU-145 and PC-3 cells [68]. 

In our study, PUFAs were administered in the form of FA lithium salts. Lithium salts, especially LiCl and Li_2_CO_3_, have already been tested and have demonstrated anticancer properties in in vitro experiments, in animal models, and in clinical studies [69]. In a study where the prostate cancer cell line LNCaP was used, LiCl exhibited significant cytotoxicity in very low concentrations (2.5 mM), and an induction of apoptosis was documented [70]. In another study, LiCl was administered on DU-145, a model also used in our experiments, combined with the chemotherapeutic agents doxorubicin and etoposide. Synergistic actions were documented; however, the IC_50_ exhibited by LiCl alone was measured to be around 20 mM after 48 h of exposure [71]. Other groups, mainly focusing on hepatocellular carcinoma and breast cancer, came up with similar results [72,73,74,75,76,77]. Another study, testing LiCl, documented the suppression of the proliferation in the ameloblastoma cell line AM-1, using a dose of 1 mM [78]. All these studies emphasize the potential of lithium as an anticancer agent, whose effects in our case were combined with the properties of GLA and other PUFAs. The lipid mixture we administered is both a carrier of lithium and polyunsaturated fatty acids, and it exhibited cytotoxicity in very low concentrations. Given that *C. elegans* FALS contained GLA, with a molecular weight of 278.43 g/mol, the dose of 60 μg/mL, which corresponds to the IC_50_, would equal about 0.2 mM, which is 25-fold lower than the Li_2_CO_3_ IC_50_ and more than 10-fold lower than the LiCl one. This dose of lithium, although it is significantly low, should be further examined for effects on the neural system and the kidneys. The low dose of lithium in the form of FALS carries all the beneficial effects of its antitumor activity, multiplied by the oxidative stress-inducing PUFAs, while simultaneously it does not load the organism with excessive amounts of the element that could lead to serious collateral effects.

Overall, the results of our study indicate that MLs rich in PUFAs and especially ω-6 fatty acids can be beneficial as anticancer formulations. GLA, being able to elevate oxidative stress levels, could be used as a synergistic agent with chemotherapies that target cancer cells and aim to induce cell death [79]. Given the elevated ROS levels already correlated with pathophysiological conditions and also being a typical aspect of the tumor microenvironment, FALS can be a promising approach. Further research must be conducted on in vivo models to further study the effects, tolerance, and toxicity of FALS on organisms; nevertheless, the utilization of microbial lipid biotechnology as a source of therapeutic agents is an emerging field with high potential and much room for improvement.

## Figures and Tables

**Table 1:** Fatty acid composition of the cellular lipids of *Cunninghamella elegans* NRRL-1393 (%, *w*/*w* of total lipids) on media composed of glucose or glycerol under nitrogen limitation in shake-flask experiments. Each experimental point is the mean value of two independent determinations (relative SE for most experimental points ≤ 15%).

Cellular Fatty Acids	C16:0	C18:0	^Δ9^C18:1	^Δ9,12^C18:2	^Δ6,9,12^C18:3
*C. elegans* on glycerol					
50 h	19.8 ± 3.1	10.5 ± 2.8	47.7 ± 4.2	12.2 ± 2.4	9.2 ± 1.8
192 h	21.4 ± 3.9	7.4 ± 1.7	48.8 ± 4.4	11.9 ± 2.1	9.5 ± 1.7
312 h	19.5 ± 2.8	5.2 ± 1.7	52.5 ± 4.9	14.8 ± 2.7	5.8
*C. elegans* on glucose					
50 h	23.2 ± 3.9	8.1 ± 1.8	47.4	11.8	8.4
192 h	23.3 ± 3.1	7.6 ± 2.8	48.1	11.0	8.8
312 h	18.6 ± 2.5	6.8 ± 1.3	50.9	15.1	7.5

**Table 2:** The optimum conditions and desirability of the response (weight of FFAs in grams).

Number	Time	Hexane	Salt	Weight of FFAs (g)	Desirability
1	60.00	15.000	1.00	0.749	0.849 selected
2	60.00	15.022	1.00	0.749	0.848
3	60.00	15.035	1.00	0.750	0.848
4	60.20	15.000	1.00	0.748	0.847

**Table 3:** IC_50_ (μg/mL) of FALS from *Cunninghamella elegans*. The values were obtained from triplicate experiments on every cell line and were analyzed using the Prism 8 software.

Cell Line	Nthy-Ori 3-1	TPC-1	K1	PC-3	DU-145
Mean IC_50_ (μg/mL)	58.81	58.60	57.89	59.21	60.69
Std. Deviation	3.063	5.358	4.468	6.855	2.862
Std. Error	1.768	3.093	2.580	3.957	1.652

**Figure A1:**
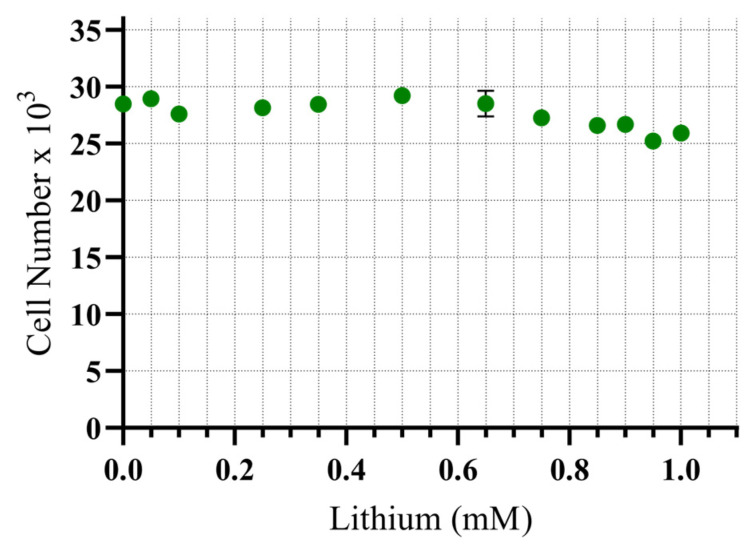
Proliferation assay of DU-145 cells. Following 48 h of incubation with increasing concentrations of Li ions, the number of remaining alive cells was determined using the crystal violet assay. The data were analyzed using Prism 8. The experiments were conducted in triplicate, and every point represents the average cell number ± SEM values.

**Figure A2:**
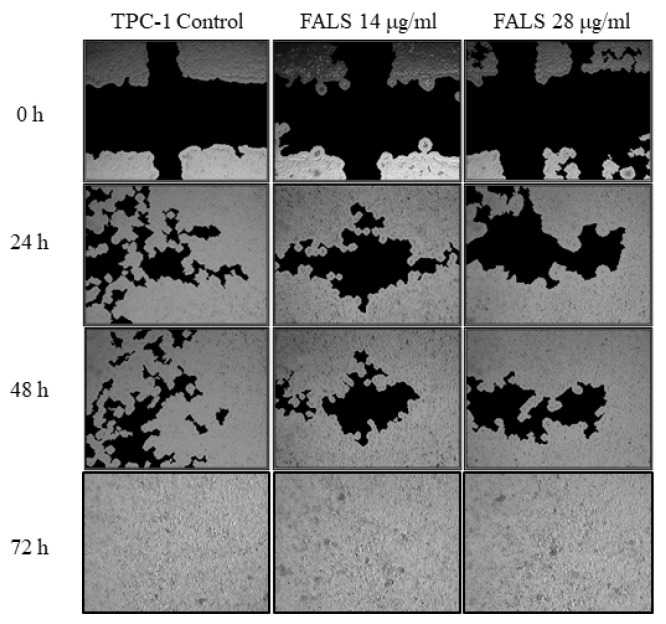
Photographs from the TPC-1 wound healing assay. Using the scratch test, we assessed the effects of FALS on the wound-healing ability of TPC-1 cells. Low doses of FALS were added (14 μg/mL and 28 μg/mL) after the wound formation, and photographs were taken every 24 h (magnification ×100). Cells supplemented only with 10% FBS were used as control samples. The data analysis was performed using the wound healing macro tool in ImageJ, which also created the shaded area wherever no cells used to be.

**Figure A3:**
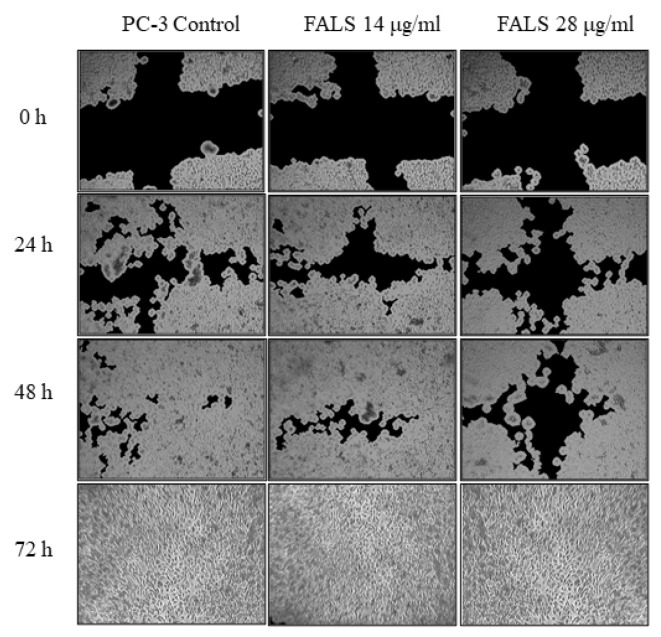
Photographs from the PC-3 wound healing assay. Using the scratch test, we assessed the effects of FALS on the wound-healing ability of PC-3 cells. Low doses of FALS were added (14 μg/mL and 28 μg/mL) after the wound formation, and photographs were taken every 24 h (magnification ×100). Cells supplemented only with 10% FBS were used as control samples. The data analysis was performed using the wound healing macro tool in ImageJ, which also created the shaded area wherever no cells used to be.

**Figure A4:**
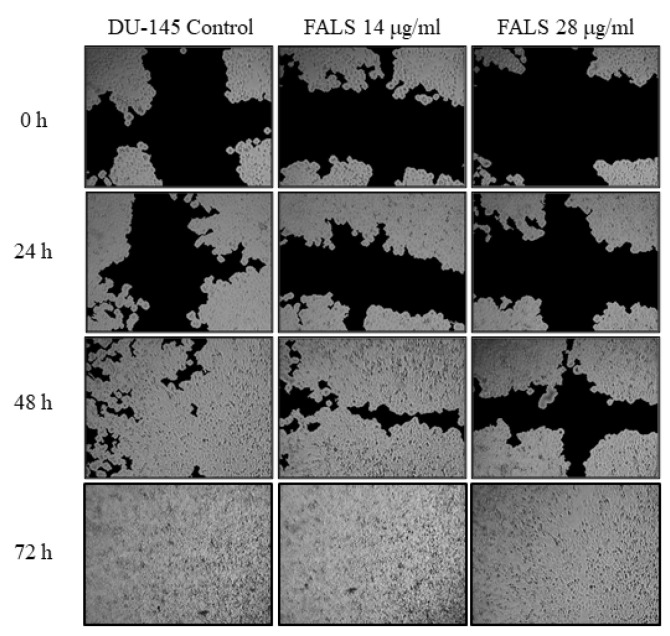
Photographs from the DU-145 wound healing assay. Using the scratch test, we assessed the effects of FALS on the wound-healing ability of DU-14 cells. Low doses of FALS were added (14 μg/mL and 28 μg/mL) after the wound formation, and photographs were taken every 24 h (magnification ×100). Cells supplemented only with 10% FBS were used as control samples. The data analysis was performed using the wound healing macro tool in ImageJ, which also created the shaded area wherever no cells used to be.

**Figure A5:**
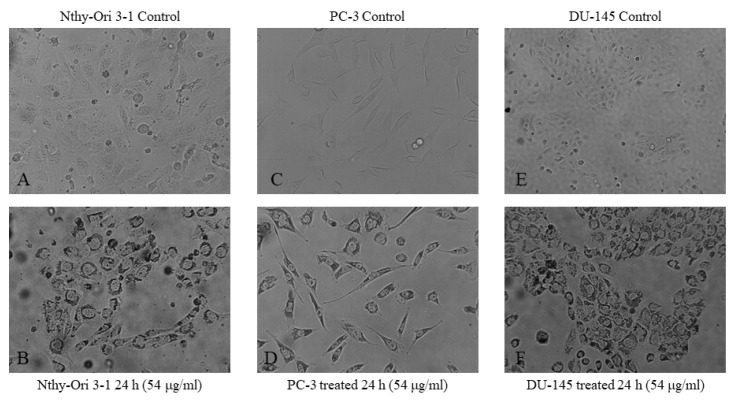
Cell morphology under a phase-contrast microscope. Following incubation with 54 μg/mL FALS for 24 h, cells grown on glass coverslips were visualized under a phase contrast microscope. All photographs were taken at ×200 magnification. (**A**) Untreated Nthy-Ori 3-1 cells; (**B**) treated Nthy-Ori cells where granulation and darker colors are visible; (**C**) untreated PC-3 cells; (**D**) treated PC-3 cells; (**E**) untreated DU-145 cells; and (**F**) treated DU-145 cells. All cell lines exhibited darker coloration, increased and abnormal size, and visible granulation.

**Figure A6:**
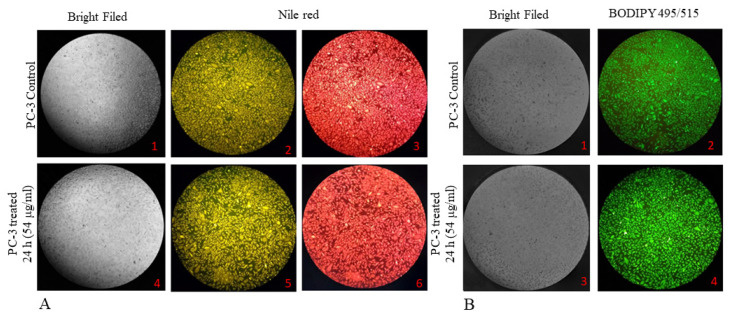
Lipid accumulation fluorescent microscope. Following incubation with 54 μg/mL FALS for 24 h, cells were grown on glass coverslips and stained with (**A**) Nile red and (**B**) BODIPY™ 505/515. All photographs were taken at ×200 magnification. (**A1**) Bright-field image of untreated PC-3 cells; (**A2**) yellow fluorescence emitted by Nile red, indicating neutral lipid and sterol content of untreated PC-3 cells; (**A3**) red fluorescence by Nile red, indicating phospholipid content of untreated PC-3 cells; (**A4**) bright-field image of treated PC-3 cells; (**A5**) yellow fluorescence emitted by Nile red, indicating neutral lipid and sterol content of treated PC-3 cells, showing increased presence; (**A6**) red fluorescence by Nile red, indicating phospholipid content of treated PC-3 cells, which is similar to that of untreated cells. (**B1**) Bright-field image of untreated PC-3 cells; (**B2**) fluorescence emitted by BODIPY™ 505/515 after staining of untreated cells; (**B3**) bright-field image of treated PC-3 cells; (**B4**) fluorescence emitted by BODIPY™ 505/515 after staining of treated cells. The increased intensity of the emitted fluorescence is typical of lipid accumulation.

**Figure A7:**
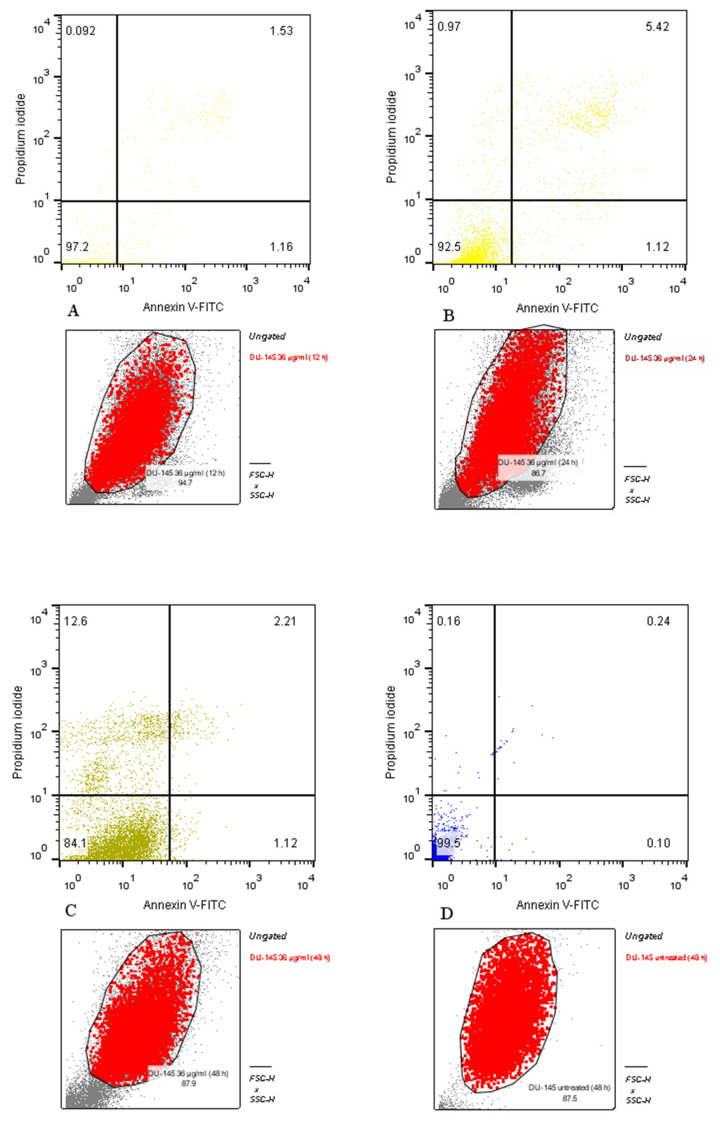

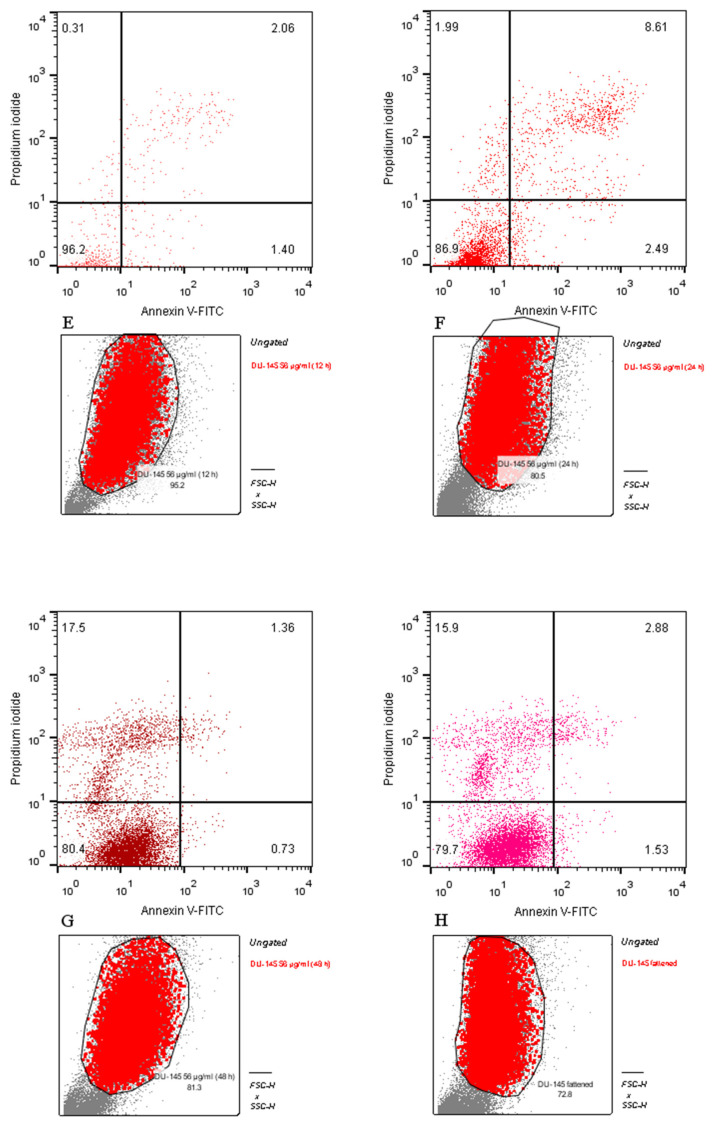
Apoptosis assays using Annexin V and propidium iodide. Cells were incubated with moderate/low (36 μg/mL) and high (54 μg/mL) doses of *Cunninghamella elegans* FALS and then trypsinized. The apoptotic/necrotic and ferroptotic portions (of detached cells) were analyzed with flow cytometry. Alive cells are shown as Annexin V-/PI-; cells in early apoptosis are represented by Annexin V+/PI-; cells in late apoptosis appear as Annexin-V+/PI+; and necrotic or ferroptotic cells are Annexin V-/PI+. (**A**) Alive PC-3 cells were used as a control sample, with a stable rate of apoptosis of about 0.5%. (**B**–**D**) The effects of the low FALS dose were assessed at 12, 24, and 48 h, and loss of cell viability down to 84% was documented. (**F**–**H**) The effects of the high dose were assessed and found to decrease cell viability by 21%. (**E**) The fattened cells were found to have a great portion of non-viable cells, despite the constant presence of FALS.

## Data Availability

We confirm that the data supporting the findings of this research are contained within the article and its appendices. Raw data supporting the results of this paper are available from G.K. and P.K., upon reasonable request.

## Appendix A

**Table A1:** Summarization of values assigned in the central composite design (CCD) experimental set-up involving three variables with one response (weight of FFAs in g) in the saponification step for fatty acid salts production of the *C. elegans* lipids.

	Variables			Response
Run	A: Time (min)	B: Hexane (mL)	C: Salt (g)	Weight of FFAs (g)
1	82.5	18.0	2.0	0.6824
2	60.0	21.0	1.0	0.7693
3	82.5	13.0	2.0	0.5876
4	82.5	18.0	2.0	0.6948
5	105.0	21.0	3.0	0.7414
6	82.5	18.0	2.0	0.6976
7	120.3	18.0	2.0	0.7906
8	82.5	23.0	2.0	0.6926
9	105.0	15.0	1.0	0.7518
10	82.5	18.0	2.0	0.7583
11	44.7	18.0	2.0	0.8732
12	82.5	18.0	0.3	0.7632
13	82.5	18.0	2.0	0.7364
14	60.0	15.0	3.0	0.6810
15	60.0	15.0	1.0	0.7475
16	105.0	21.0	1.0	0.7290
17	82.5	18.0	3.7	0.6722
18	82.5	18.0	2.0	0.6739
19	105.0	15.0	3.0	0.7558
20	60.0	21.0	3.0	0.7429

## Appendix B

In order to conclude whether the cytotoxicity exhibited by the FALS was a result of the Fas or the lithium ions, we administered lithium (as LiOH diluted in PBS) at concentrations similar to the FALS and assayed the cell proliferation rate after 48 h of incubation. The concentrations applied did not interfere with the cells’ ability to proliferate (Figure A1). Therefore, the lithium effects at such low concentrations were considered negligible.

To study the effects of FALS on different types of cell motility, we organized a series of experiments that utilized the Boyden chamber assay as well as the scratch test/wound healing assay. Our scratch test assays were performed in six-well microplates where artificial wounds were made (after the formation of cell monolayers) using a P200 tip. In preliminary experiments, the cells were assayed in the absence of FBS; nevertheless, FALS fully inhibited wound closure, and extensive apoptosis was observed. Subsequently, we supplemented the cells with 10% FBS, and this led to a complete wound closure within 72 h in all three cell lines (Figure A2, Figure A3 and Figure A4). FALS were observed to slow down the process since their cytostatic effects were proportional to the administered dose.

## Appendix C

To visualize morphological differences as a result of incubation with FALS, we cultured cells on glass coverslips and viewed them under a phase-contrast microscope (Figure A5). Both normal (Nthy-Ori 3-1) and cancerous (PC-3 and DU-145) cells exhibited the same phenotype: enlarged cell size, atypical morphology, and intensive granulation in the cytosolic area. The granules are thought to be vesicles inside the endoplasmic reticulum (ER), structures known as lipid droplets that are formed after the accumulation of a vast amount of neutral lipids.

## Appendix D

To investigate whether incubation with FALS would increase the lipid content inside the cells, we cultured cells on glass coverslips, stained them with lipid-specific fluorescent dyes, and viewed them under a fluorescent microscope (Figure A6). Nile Red was used to observe both neutral lipids (using a yellow filter) as well as phospholipids (using a red filter) that emit light at longer wavelengths. BODIPY™ 505/515 was used to verify these results.

## Appendix E

In this part, we provide the flow cytometry data, showing the different populations after staining with Annexin V-FITC conjugated and propidium iodide. Double-negative populations are alive cells, and double-positive populations are cells undergoing apoptotic death. The Annexin V+/PI- population is categorized as cells in the early stages of apoptosis, where the cell membrane still maintains its integrity, while the Annexin V-/PI+ population are cells undergoing cell death but of a non-apoptotic nature. In our result section, those cells are referred to as necrotic or ferroptotic.
